# SO_2_ Donors and Prodrugs, and Their Possible Applications: A Review

**DOI:** 10.3389/fchem.2018.00559

**Published:** 2018-11-16

**Authors:** Wenyi Wang, Binghe Wang

**Affiliations:** Department of Chemistry and Center for Diagnostics and Therapeutics, Georgia State University, Atlanta, GA, United States

**Keywords:** sulfur dioxide, gasotransmitters, donors, prodrugs, applications

## Abstract

SO_2_ is widely recognized as an air pollutant and is a known cause of acid rain. At a sufficiently high level, it also causes respiratory diseases. A much lesser known side of SO_2_ is its endogenous nature and possible physiological roles. There is mounting evidence that SO_2_ is produced during normal cellular metabolism and may possibly function as a signaling molecule in normal physiology. The latter aspect is still at the stage of being carefully examined as to the validity of classifying SO_2_ as a gasotransmitter with endogenous signaling roles. One difficulty in studying the biological and pharmacological roles of SO_2_ is the lack of adequate tools for its controllable and precise delivery. Traditional methods of using SO_2_ gas or mixed sulfite salts do not meet research need for several reasons. Therefore, there has been increasing attention on the need of developing SO_2_ donors or prodrugs that can be used as tools for the elucidation of SO_2_'s physiological roles, pharmacological effects, and possible mechanism(s) of action. In this review, we aim to review basic sulfur chemistry in the context of sulfur signaling and various chemical strategies used for designing SO_2_ donors. We will also discuss potential pharmacological applications of SO_2_ donors, lay out desirable features for such donors and possibly prodrugs, analyze existing problems, and give our thoughts on research needs.

## Introduction

SO_2_ has long been recognized as an air pollutant produced by fossil fuel combustion and volcano eruptions. Upon oxidation and/or hydration, SO_2_ can serve as a precursor of acid species such as H_2_SO_3_ and H_2_SO_4_, which cause acidification of rain water, and thus acid pollution. In terms of toxicology, overexposure to SO_2_ may induce respiratory tract irritation and damage to many organs (Meng, 2003; Meng et al., 2004; Iwasawa et al., 2009; Sang et al., 2010). Besides forming acid which causes mucous irritation, the mechanism of SO_2_'s toxicity is generally thought to involve the oxidation process of its derivatives (Mathew et al., 2011). For example, it has been demonstrated that the DNA damage effect of SO_2_ can be induced by ${\text{SO}}_{3}^{\text{-}}$ and ${{\text{SO}}_{4}}^{•-}$ radicals produced by autoxidation of sulfite (SO_3_^2−^) (Shi and Mao, 1994; Muller et al., 1997). However, as a matter of fact, SO_2_ and its derivatives have a long history of being applied in the food industry as preservatives. Moreover, endogenous SO_2_ can be produced enzymatically through the metabolism of sulfur-containing amino acids or non-enzymatically through oxidation of H_2_S in neutrophils under oxidative stress (Mitsuhashi et al., 2005; Mathew et al., 2011). A recent review by Huang et al. considered SO_2_ as the fourth gasotransmitter after NO, CO, and H_2_S (Huang et al., 2016). By the same token, it is hard to imagine that O_2_ and CO_2_ do not have regulatory roles and should not be considered as gasotransmitters as well. Indeed, sensing for O_2_ and CO_2_ is essential for their regulatory roles and have been extensively studied (Kaelin and Ratcliffe, 2008; Lopez-Barneo et al., 2008; Cummins et al., 2014). Therefore, one may consider the gasotransmitter family having at least six members.

In recent years, accumulating evidence demonstrates that SO_2_ possesses many bioactivities, especially in the cardiovascular system (Liu et al., 2010; Wang X. B. et al., 2014). Besides the biological effect SO_2_ demonstrated under physiological concentration, it has been shown that abnormal levels of SO_2_ are related to many pathological conditions such as hypertension and pulmonary hypertension (Huang et al., 2016). Its regulatory effects in the cardiovascular system may have therapeutic potential and thus require more detailed studies for its mechanism of activity (Wang et al., 2015).

### SO_2_ chemistry

**Figure 1:**
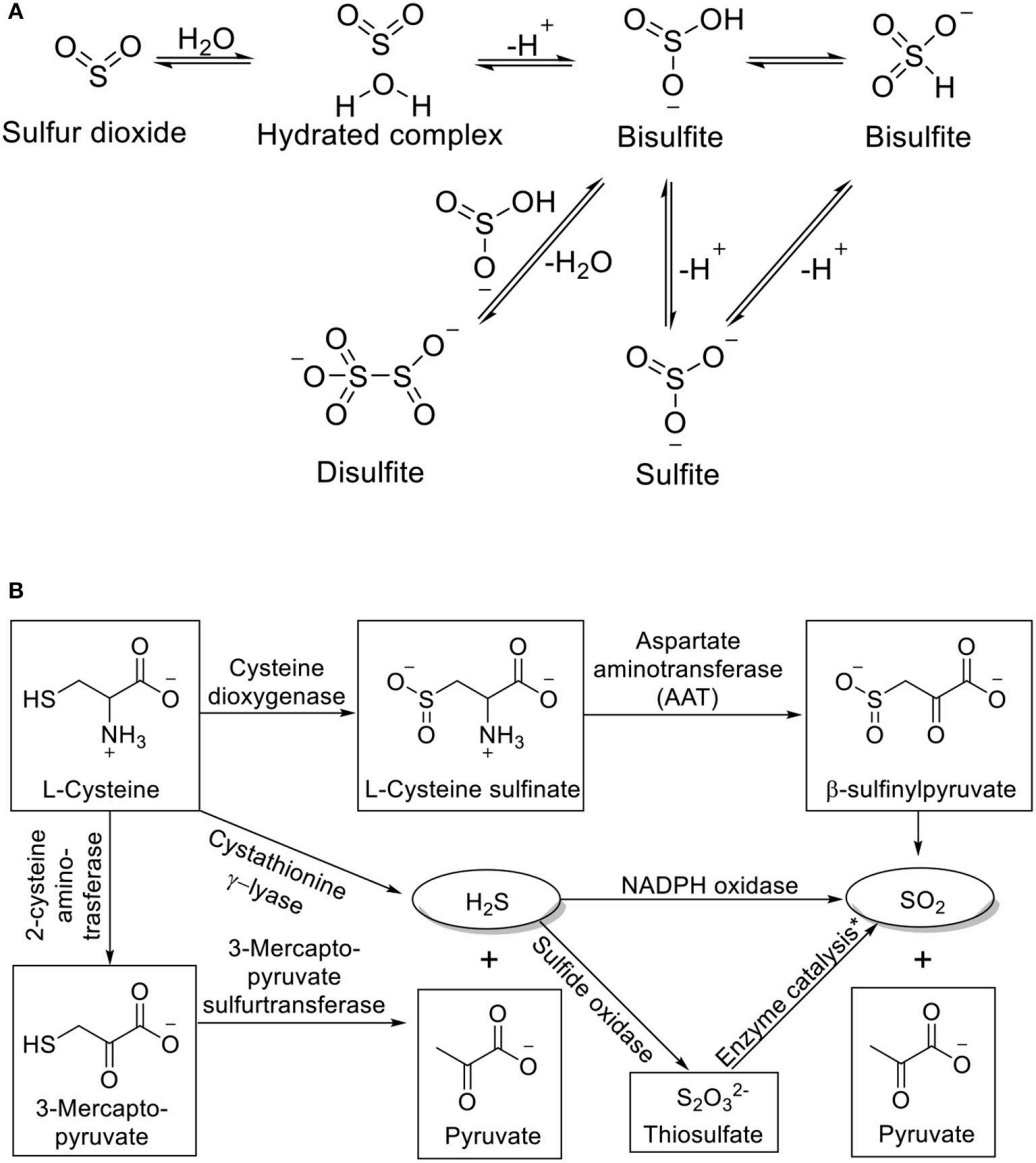
**(A)** Interconversion of SO_2_ derivatives and **(B)** Endogenous production of SO_2_. ^*^Process catalyzed by thiosulfate sulfurtransferase or glutathione-dependent thiosulfate reductase.

Much of the recent rise in interest for SO_2_ originates from its possible roles as an endogenous signaling molecule. As a gaseous molecule, SO_2_ has a Henry's constant of 1.23 mol kg^−1^ bar^−1^ (Goldberg and Parker, 1985). It can readily dissolve in water and form a hydrated SO_2_ complex (SO_2_·H_2_O), which in turn undergoes first and second dissociation to give bisulfite (${\text{HOSO}}_{2}^{\text{-}}$ or ${\text{HSO}}_{3}^{\text{-}}$) and sulfite (SO_3_^2−^) ions (Tolmachev and Scherson, 1999; Voegele et al., 2004; Townsend et al., 2012). The first and second pK_a_s of SO_2_·H_2_O are 1.81 and 6.97. Accordingly, it can be calculated that under physiological pH, the bisulfite and sulfite are formed in a molar ratio of about 1: 3. Dimerization of bisulfite would form disulfite (S_2_${\text{O}}_{5}^{\text{-}}$). The interconversion of these species is summarized in Figure 1A. Due to the abundance of water in the biological system, all the species exist in an equilibrium. Therefore, it will be hard to isolate one species out and study its sole effect; and it is important to study SO_2_ and its derivatives as a whole. Besides hydration, another important aspect of SO_2_ chemistry is its redox chemistry, which is much more complex in a biological setting. SO_2_ and its derivatives are interconnected with many other sulfur species through enzymatic or non-enzymatic processes. Generally speaking, SO_2_ is produced as an intermediate of metabolism of sulfur-containing compounds and can be oxidized further to generate other sulfur species. For example, SO_2_ can be produced from L-cysteine through sequential catalysis of cysteine dioxygenase and aspartate aminotransferase (AAT); oxidation of sulfite through sulfite oxidase can produce sulfate; H_2_S can be generated from L-cysteine by cystathionine β-synthase, and further oxidation by NADPH oxidase would generate SO_2_; under ambient conditions, more reactive species such as ${\text{SO}}_{3}^{\text{-}}$ and ${\text{SO}}_{4}^{\text{-}}$ can be produced through autoxidation of sulfite (Muller et al., 1997; Huang et al., 2016; Bora et al., 2018).

Taken together the chemistry of SO_2_ and current reports about SO_2_'s biological activity, one may see some contradictory results. On one hand, SO_2_ and its derivatives seem to be toxic intermediates produced in sulfur metabolism. The high oxidation state of endogenous SO_2_ produced by oxidation of sulfur species at lower oxidation states (e.g., cysteine, H_2_S) can pose oxidative stress on cells and tissue, causing oxidative damage (Meng, 2003). Moreover, deficiency of sulfite oxidase, which catalyzes the oxidation of sulfite to sulfate as the last oxidative step of sulfur metabolism, is known to result in fatal neurological symptoms (Karakas and Kisker, 2005; Wang S. S. et al., 2017). On the other hand, the potential for further oxidation allows SO_2_ and its derivatives to function as an antioxidant. Indeed, SO_2_ plays a protective role against oxidative stress under certain circumstances (Liang et al., 2011; Wang et al., 2011a; Jin et al., 2013; Chen et al., 2015). For example, Chen et al. have demonstrated that endogenous SO_2_ offers protection against oleic acid-induced acute lung injury by suppressing oxidative stress (Chen et al., 2015). Also, SO_2_ preconditioning was shown to be effective in preventing ischemia-reperfusion injury in the cardiovascular system (Wang et al., 2011a). It is nonetheless clear that the chemical reaction to go from SO_2_ to SO_3_ is only a 2-electron process. Thus, the scavenging capacity of SO_2_ is far lower than that of H_2_S, which can give 6 electrons to go to the oxidation state of SO_2_ and 8 electrons to that of SO_3_. The dual role of SO_2_ and its “inefficiency” as antioxidant makes us wonder what the reason would be for SO_2_ to be used as an antioxidant by nature. Does SO_2_ perform its “antioxidant” role through direct reduction/scavenging of an oxidizing species or through regulatory functions that make cells more resilient under an oxidizing environment? The inter-conversion rates among sulfur species are also very important in considering the meaning of using SO_2_ as a signaling molecule. Enzymatic processes not only provide more precise control of substrate concentration compared with the non-enzymatic process but also offer potential targets for regulation. In addition, bisulfite and sulfite are strong nucleophiles and such properties may contribute to their biological activities. For all these reasons and more, it is very important to understand the chemistry issues when studying its biology and developing its donors and prodrugs. However, such relationships are very convoluted that makes deciphering the precise role of SO_2_ very hard, given that the enzymes and reaction kinetics involved are not clear.

For a signal molecule to function, the existence of relevant biological target(s) is a critical question. For small organic molecules, proteins, peptides, lipids, and others, binding to a macromolecular target is often the mechanism of action. The issue is nevertheless more challenging for gasotransmitters. There is a well-established mechanism(s) of action at the molecular level for nitric oxide (NO), hydrogen sulfide (H_2_S) (Zheng et al., 2018) and carbon monoxide (CO) (Ji and Wang, 2018). Specifically, NO can bind to a heme moiety at the distal site of a soluble form of guanylyl cyclase, and thus activates guanylyl cyclase, leading to elevated levels of cGMP (Schmidt et al., 1993). Hydrogen sulfide in its persulfide form is known to be responsible for protein persulfidation, which is a well-established regulatory process (Yu et al., 2018). For CO, there is also strong evidence that binding to heme and other metal-containing biomolecules is a critical first step (Knauert et al., 2013; Ji and Wang, 2018). For example, CO can bind a soluble form of guanylyl cyclase much the same way as NO, except with decreased ability to activate the cyclase. CO is also reported to inhibit cytochrome C oxidase leading to increased production of ROS, which in turn leads to the production of the persulfide form of glutathione (GSSG) (Brown and Piantadosi, 1990; Zhang and Piantadosi, 1992; Alonso et al., 2003). GSSG is known to facilitate protein glutathionylation, a known form of protein post-translational modification, and plays a regulatory role. With SO_2_, molecular level studies have been scarce, though various regulation effects of SO_2_ and its derivatives on other biological molecules have been observed, especially in the cardiovascular system (Huang et al., 2016). However, the binding targets of SO_2_ or its derivatives are not yet clear. It is not hard to envision that the equilibrium between SO_2_ and other sulfur species would play an important role in maintaining the intracellular redox balance. Therefore, enzymes involved in generating related species may serve as potential binding targets. Moreover, the varied concentration of SO_2_ in different tissue hints that SO_2_ may have tissue-specific targets (Du et al., 2008). Furthermore, as a strong nucleophile and Lewis base, bisulfite's interaction with certain metal-containing proteins (e.g., sulfite oxidase) and reaction with electrophiles could play a role in regulating the biological functions of these molecules (Cohen et al., 1971), With such knowledge, SO_2_ donors and prodrugs with desirable properties will help us understand the molecular mechanism of SO_2_'s action.

### Endogenous production and physiological effects of SO_2_

SO_2_ can be produced endogenously through several pathways (Figure 1B). In the AAT pathway, L-cysteine is first oxidized to L-cysteine sulfinate by cysteine dioxygenase (CDO). AAT then catalyzes the transamination between L-cysteine sulfinate and α-ketoglutarate to form β-sulfinylpyruvate. The spontaneous decomposition of the latter would yield SO_2_ and pyruvate (Singer and Kearney, 1956). SO_2_ can also be produced from the oxidation of H_2_S, another metabolite of sulfur-containing amino acids. The oxidation could be directly through NADPH oxidase in activated neutrophils, or through oxidation by sulfide oxidase followed by thiosulfate sulfurtransferase or glutathione-dependent thiosulfate reductase (Kamoun, 2004; Mitsuhashi et al., 2005; Qu et al., 2008). Zhang et al. have shown that the endogenous production of SO_2_ is closely related to endogenous H_2_S. Endogenous H_2_S can inhibit endothelial SO_2_ production through suppressing AAT activity, while impaired H_2_S production pathway would lead to upregulation of SO_2_ production (Zhang et al., 2018). The excretion of SO_2_ is through hydration and oxidation by sulfite oxidase to sulfate followed by renal clearance to urine (Stipanuk, 1986).

The most profound effect of SO_2_ under physiological conditions was found in the cardiovascular system. Du et al. tested various vascular tissues and found the highest SO_2_ content in the aorta (Du et al., 2008). Using a mixture of bisulfite/sulfite salt as an SO_2_ source, a slight relaxation effect was observed on isolated rat aortic rings while a concentration-dependent relaxation was observed with higher dosages. The same effect was also observed using SO_2_ gas and SO_2_ gas solution as later demonstrated by Meng et al. (2003, 2009). Such result indicates the role SO_2_ plays in maintaining normal vascular tone and blood pressure. SO_2_ was also found to have a negative inotropic effect as observed on isolated perfused rat heart, which in turn affects the heart rate and left ventricular developed pressure (Zhang and Meng, 2012). Besides in the cardiovascular system, SO_2_ was also found to be involved in lipid metabolism, possibly due to its oxidant effect (Haider, 1985; Lovati et al., 1996). More interestingly, aligned with the well-known antimicrobial activity of SO_2_ and its derivatives, Nojima and colleagues have demonstrated that sulfite is a neutrophil mediator of host defense that *in vivo* administration of lipopolysaccharide (LPS) would trigger sulfite release from neutrophil and increase serum sulfite concentration in rats (Mitsuhashi et al., 1998).

### Pathophysiological effect of SO_2_

Many research papers and excellent reviews have been published in recent years addressing the pathophysiological effect of SO_2_ (Wang X. B. et al., 2014; Huang et al., 2016). Here we give a brief summary to provide a background for readers to understand the therapeutic potential and future application of SO_2_.

Compared with the SO_2_'s therapeutic potential, the toxicity of SO_2_ is more widely accepted by the public; and is likely to be attributed to SO_2_'s oxidative property. Meng has suggested that SO_2_ as a oxidative agent can cause lipid peroxidation and changes anti-oxidation status in multiple organs (Meng, 2003). Chronic exposure to SO_2_ was also found to promote atherosclerosis (Bruske et al., 2011). Controversially, in atherosclerosis rats, a decreased level of plasma and aortic SO_2_ content, as well as AAT activity, was reported by Li et al. (2011). Further, treatment of SO_2_ derivatives could diminish the size of atherosclerotic plaques in rat coronary artery. Under certain physiological or pathological conditions, the regulation of endogenous SO_2_ level may vary, resulting from or contributing to the formation of the pathological condition.

The fluctuation of endogenous SO_2_ has been observed in several animal disease models and human. Elevated level of serum sulfite content was found in patients with chronic renal failure and pneumonia (Kajiyama et al., 2000; Mitsuhashi et al., 2004). Corresponds with afore mentioned LPS stimulated sulfite release stimulated by LPS, the serum level of SO_2_ was found increased significantly in pediatric patients with acute lymphoblastic leukemia with bacteria inflammation, which is likely a defensive mechanism indicating SO_2_'s anti-inflammatory role (Wu et al., 2014).

SO_2_'s pathophysiological roles have been widely studied in cardiovascular disease models. In spontaneous hypertensive rats (SHRs), a significantly lower level of SO_2_ and AAT activity was observed in serum and aorta (Zhao et al., 2008). In a study conducted on children with congenital heart disease (CHD), serum SO_2_ level showed a negative relationship with pulmonary hypertension. For example, in the CHD group with severe pulmonary hypertension the SO_2_ concentration was only half of that of the group without pulmonary hypertension (Yang et al., 2014). Similarly, in rats under hypoxic conditions, a decreased plasma and lung tissue SO_2_ level was observed simultaneously with pulmonary hypertension, pulmonary vascular structural remodeling, and increased vascular inflammatory response (Sun et al., 2010). In rat model with monocrotaline-induced pulmonary vascular collagen remodeling, SO_2_ content and AAT activity were significantly increased (Yu et al., 2016). Meanwhile, in rats with monocrotaline (MCT) induced pulmonary hypertension, higher SO_2_ level, AAT expression and activity were observed compared to the control group, indicating a protective role of endogenous SO_2_ (Jin et al., 2008). Additionally, in rat myocardial ischemia-reperfusion (I/R) models, AAT1 protein expression showed a significant decrease (Wang et al., 2011b). In isoproterenol induced myocardial injury rat model, the SO_2_/AAT pathway also showed a down regulation (Liang et al., 2011). Subjecting such animal models to treatment or pretreatment with SO_2_ derivatives or SO_2_ gas led to alleviation of the pathology, indicating a strong therapeutic potential of SO_2_.

## SO_2_ donors and prodrugs

For biological assessment, SO_2_ gas and mixed sulfite salts (NaHSO_3_ and Na_2_SO_3_ in 1:3 molar ratio) have been most widely used as sources of SO_2_. SO_2_ gas was used for the preparation of stock solution or administration to small animals by inhalation. The dosage can be controlled through partial pressure. In such applications, using gaseous SO_2_ requires special laboratory equipment and settings as well as prolonged exposure time at low concentration to avoid acute toxicity or irritation. Neither does it offer precise dosage control nor future prospect of human use. The second method of using mixed sulfite salts to generate SO_2_ depends on the complex kinetics of the dynamic equilibrium between gaseous SO_2_ and its hydrated derivatives (Figure 1A). Using such donors lacking controllable release rate cannot mimic endogenous production of SO_2_ and may lead to transient effect and overdosing. The quick renal clearance rate may also limit the efficiency of using mixed salt as SO_2_, especially when prolonged exposure at low dosage is required. Moreover, the efficiency of SO_2_ delivery to cell or tissue can hardly be guaranteed and may result in irreproducible experiment results. According to Meng and co-workers, gaseous SO_2_ may have a much stronger vasorelaxant effect compared with mixed sulfite salts (Meng et al., 2009). Another way of donating SO_2_ is through overexpression of two isoenzymes of AAT (Liu et al., 2014). AAT is an upstream enzyme in the SO_2_ generation pathway, which can catalyze the transamination of L-cysteine sulfinate to form β-sulfinylpyruvate and generate SO_2_. Therefore, the activity of AAT may directly influence SO_2_ production. Vascular smooth muscle cells (VSMCs) transfected with AAT1 or AAT2 plasmids showed suppressed serum-induced proliferation, while AAT1 or AAT2 knocked-down VSMCs showed exacerbated serum-induced proliferation. Such approaches could serve as a powerful research tool in cell-based assays. Nevertheless, the same kind of studies at the tissue, organ, or animal level would require much more complicated work (Naldini, 2015). The lack of reliable donors that can generate SO_2_ in a controllable manner has hampered the delineation of SO_2_'s precise biological and regulatory roles. To overcome these obstacles, SO_2_ donors and prodrugs with different triggering mechanisms have been developed in recent years. Below, we have summarized the development of SO_2_ prodrugs with specific emphases on their design principles, release mechanism(s), and biological applications.

### Thiol-activated SO_2_ prodrugs

**Scheme 1:**
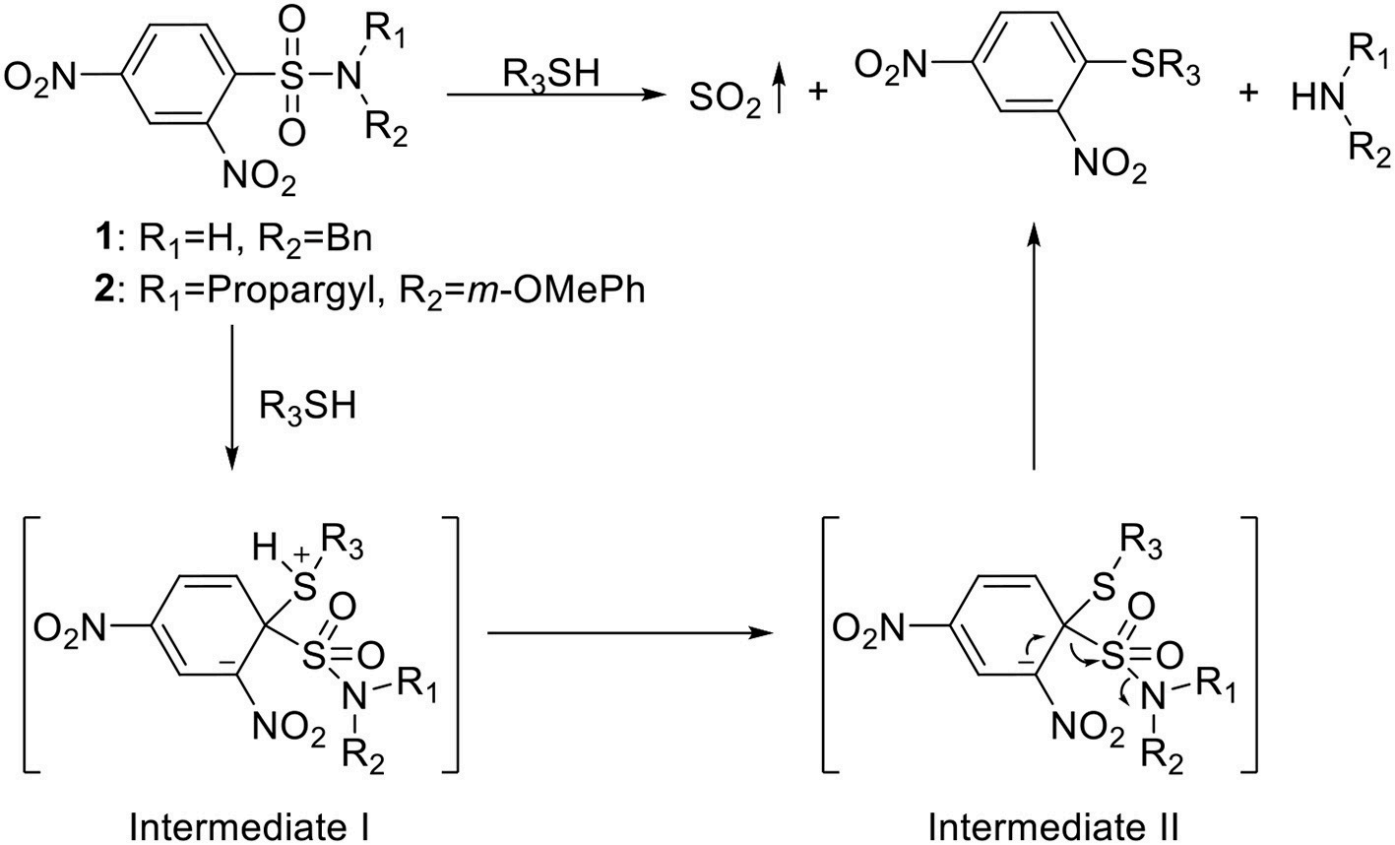
Thiol-activated SO_2_ prodrugs.

Chakrapani's group pioneered in the work of developing SO_2_ prodrugs. They first developed a series of 2,4-dinitrophenylsulfonamide compounds as prodrugs that release SO_2_ upon thiol triggering for antimycobacterial purpose (Scheme 1) (Malwal et al., 2012b). 2,4-Dinitrophenylsulfonamide was previously employed in thiol-detection systems, which produces SO_2_ as a byproduct upon decomposition. In their design, mycothiol (MSH), which exists in *Mycobacterium tuberculosis* (*Mtb*) in millimolar concentrations, would serve as the thiol source to trigger the release. Depleting thiols and generating SO_2_ at the same time would disrupt cellular redox equilibrium and damage biomacromolecules, causing *Mtb* growth inhibition. The first batch of 17 compounds was synthesized with varied substitutions on both the amino group and the sulfonyl group through simple substitution reactions using the corresponding amines and sulfonyl chlorides. The compounds were incubated in pH 7.4 buffer with cysteine and tested of SO_2_ release by a *p*-rosaniline-based assay. Release rates were measured by ion chromatography (IC) with a conductivity detector. Antimycobacterial activity of the compounds against *Mtb* was quantified as minimum inhibitory concentrations (MICs, the minimum concentration required to inhibit 99% of bacterial growth). Eleven compounds containing the 2,4-dinitrophenylsulfone (DNs) moiety showed SO_2_ release within 30 min and demonstrated greater antimycobacterial activity than the non-releasing compounds, indicating a correlation between SO_2_ release and *Mtb* inhibition. The release mechanism was proposed to go through Jackson-Meisenheimer complex intermediates to produce SO_2_, benzylamine, and 2,4-dinitrophenylthioether (Scheme 1). *N*-Benzyl-2,4-dinitrophenylsulfonamide (**1**, Scheme 1) showed 100% SO_2_ release within 30 min (estimated half-life ~2 min) and was found to have the best *Mtb* inhibitory potency with a MIC of 0.15 μM, which is lower than the clinically used agent isoniazid (0.37 μM) tested under the same conditions. Moreover, compound **1** showed no cytotoxicity at such concentration toward human embryonic kidney 293 cells (HEK) (IC_50_ = 7 μM). Further studies of the relationship between SO_2_ release rates and MICs on 6 compounds (*t*_1/2_ = 2~63 min) with similar clog *P*-values found a good correlation between fast SO_2_ release rates and inhibitory potency.

To better understand this relationship as well as the structure-activity relationship (SAR), the same group synthesized a total of 19 structural analogs of compound **1** with varied amino substitutions and comparable clog*P* values (Malwal et al., 2012a). The results showed only decreased SO_2_ release rates and MICs compared with compound **1**, regardless of modifications made. Seven compounds with potent MICs (highest MIC = 2.13 μM) were found better than clinically used drugs ethambutol and pyrazinamide tested under the same conditions. According to Spearman rank correlation analysis, a correlation between MIC and SO_2_ yield after 5 min was established that higher SO_2_ yield is related to the lower MIC.

This strategy was further tested on methicillin sensitive *Staphylococcus aureus* (MSSA, Gram+), methicillin-resistant *S. aureus* (MRSA, Gram+), *Enterococcus faecalis* (*E. faecalis*, Gram+), and *Escherichia coli* (*E. coli*, Gram-). (Pardeshi et al., 2015) A library of 38 2,4-dinitrophenylsulfonamides (including compound **1**) was synthesized, among which 12 compounds were in “dimerized” forms. All compounds were shown to be able to release SO_2_ under physiological conditions with different rates. Compound **2** (Scheme 1) showed good MICs of 4 μg/mL (~10 μM) against both strains of *S. aureus* and 8 μg/mL MIC against *E. faecalis*. However, no compound was found to be effective against *E. coli*, possibly due to the permeation barrier presented by the outer membrane of Gram-negative bacteria in general (Nikaido, 1998). The inhibitory mechanism remains to be determined since evidence suggests that the SO_2_ generating ability of these compounds is not the sole reason responsible for the inhibitory effect. The capability of the compounds to permeate cells and deplete thiols may as well affect the inhibitory effect since thiols in presence of oxidative stress can be primarily oxidized to disulfide species. Indeed, compound **2** showed much better capacity depleting intracellular thiols compared with compounds with poor inhibitory activity. Furthermore, compound **2** was also capable of inhibiting several patient-derived MRSA strains with MIC values of 2~4 μg/mL. Cytotoxicity was also assessed against A549 lung carcinoma cells. A GI_50_ of 27 μM and a selectivity index (GI_50_/MIC) of 5.2 were determined.

Besides antibacterial applications, compound **1** has been extensively used as an intracellular SO_2_ source in the validation of utility of many SO_2_ probes in biological settings (Liu et al., 2015, 2016; Wang et al., 2016; Xu et al., 2016; Li et al., 2017). Depletion of thiol with thiol scavenger (*N*-ethylmaleimide, NEM) would easily disable SO_2_ release from the prodrug, offering a good method for conducting control experiments.

### Thermally activated SO_2_ prodrugs

**Scheme 2:**
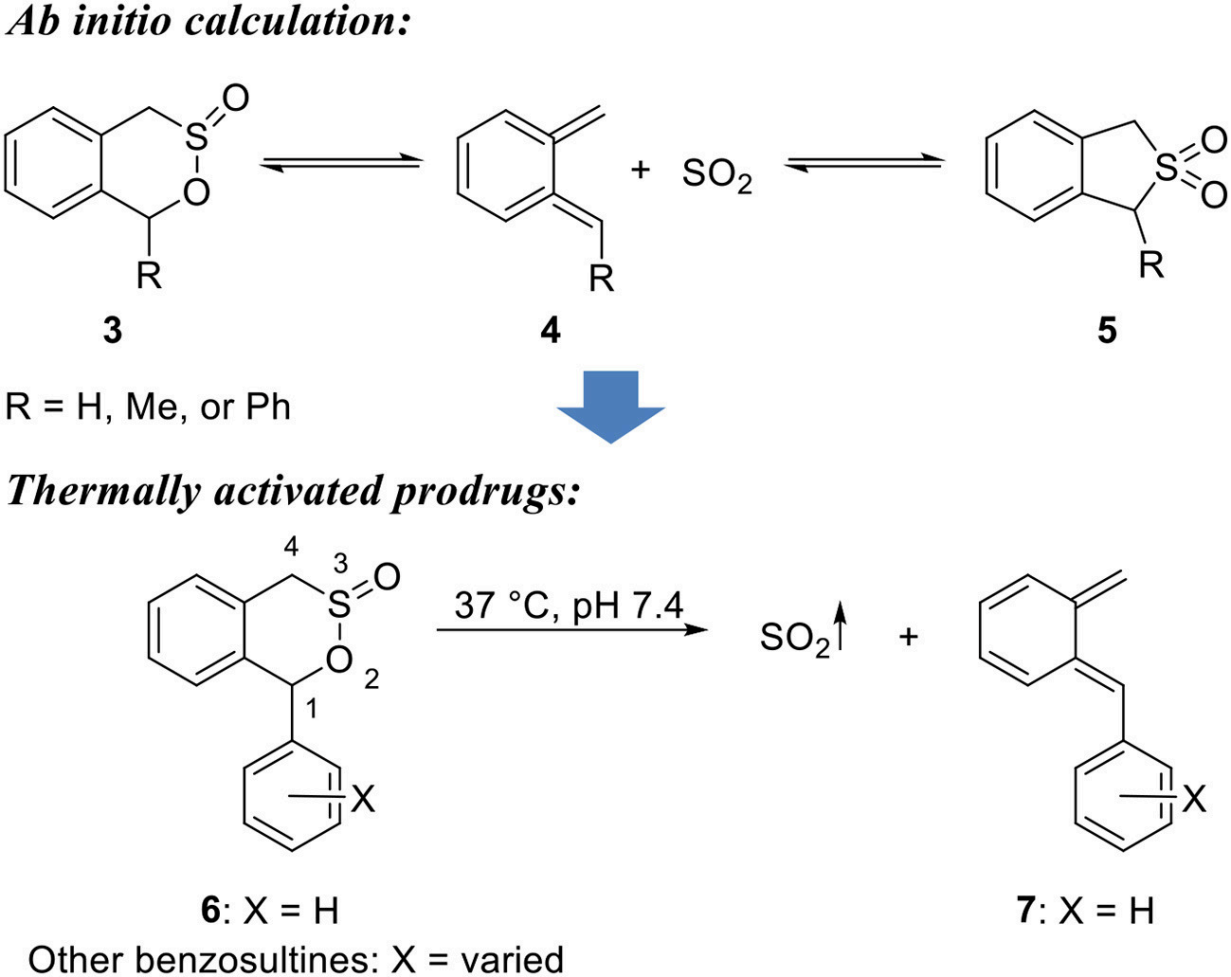
Thermally activated retro-Diels-Alder reaction-based SO_2_ prodrugs.

Another class of SO_2_ prodrugs developed by Chakrapani's groups uses the benzosultine scaffold (Scheme 2) (Malwal et al., 2013). By design, such prodrugs would release SO_2_ through a thermal retro-Diels-Alder reaction. The substitutions on the 1-position would allow reactivity tuning and thus cycloreversion at physiological temperature. As such, benzosultine and 1-substituted (Me, Ph) benzosultines were subjected to *ab initio* calculations. Reaction energies (Δ*E*_rxn_), Gibbs free energies (Δ*G*_rxn_), and Gibbs free-energy barriers (Δ*G*^‡^) of different conformations of the benzosultines and sulfones to produce dienes and SO_2_ were calculated to predict the stable conformations and favorable reaction paths (Scheme 2). Results showed that, compared with benzosultine, 1-substituted benzosultines have lower Gibbs energy barriers (Δ*G*^‡^) and would be expected to have enhanced cycloreversion rates. Besides, phenyl substitution (**6**) would further reduce cycloreversion barrier than methyl substitution, leading to faster reactions at the same temperature. All three compounds were synthesized and tested under varied thermal conditions. Compound **6** was shown to be an active SO_2_ donor at physiological pH and 37°C with a release rate constant *k* of 0.0140 min^−1^, while the other two compounds were found to be unreactive under similar conditions. Moreover, calculations predicted conversion of 1-phenyl-benzosultine (**3**) to form 1-phenyl-benzosulfone (**5**) as a more stable form (Scheme 2). This prediction was confirmed by experiments. When compound **6** was incubated in MeCN solution at 37°C, it was fully converted to 1-phenyl-benzosulfone (**7**), presumably through a retro-Diels-Alder reaction followed by *in situ* cheletropic addition of SO_2_. Based on this result, the authors further developed UV-light sensitive SO_2_ donors based on the sulfone structure, which is discussed in the next section. The utility of compound **6** as an SO_2_ prodrug was demonstrated using a DNA cleavage assay. Compound **6** showed the same potency as mixed sulfite salts in cleaving plasmid DNA. 1-Aryl-benzosultines with varied substitution groups were prepared to further explore the tunability of the reaction rate. These compounds showed release half-lives ranging from 10 to 68 min at pH 7.4 in 30–40% MeCN/PBS mixture at 37°C. According to Hammett plot of rate constants, the substitution groups were found to have a weak electronic effect on the reaction rate that electron-donating group would accelerate the reaction. However, none of these compounds showed the complete release of SO_2_, with maximal SO_2_ yield ranging from 59 to 89%. This incomplete conversion was thought to be caused by decomposition of sultine through uncharacterized process(es).

### Photochemically activated SO_2_ prodrugs

**Scheme 3:**
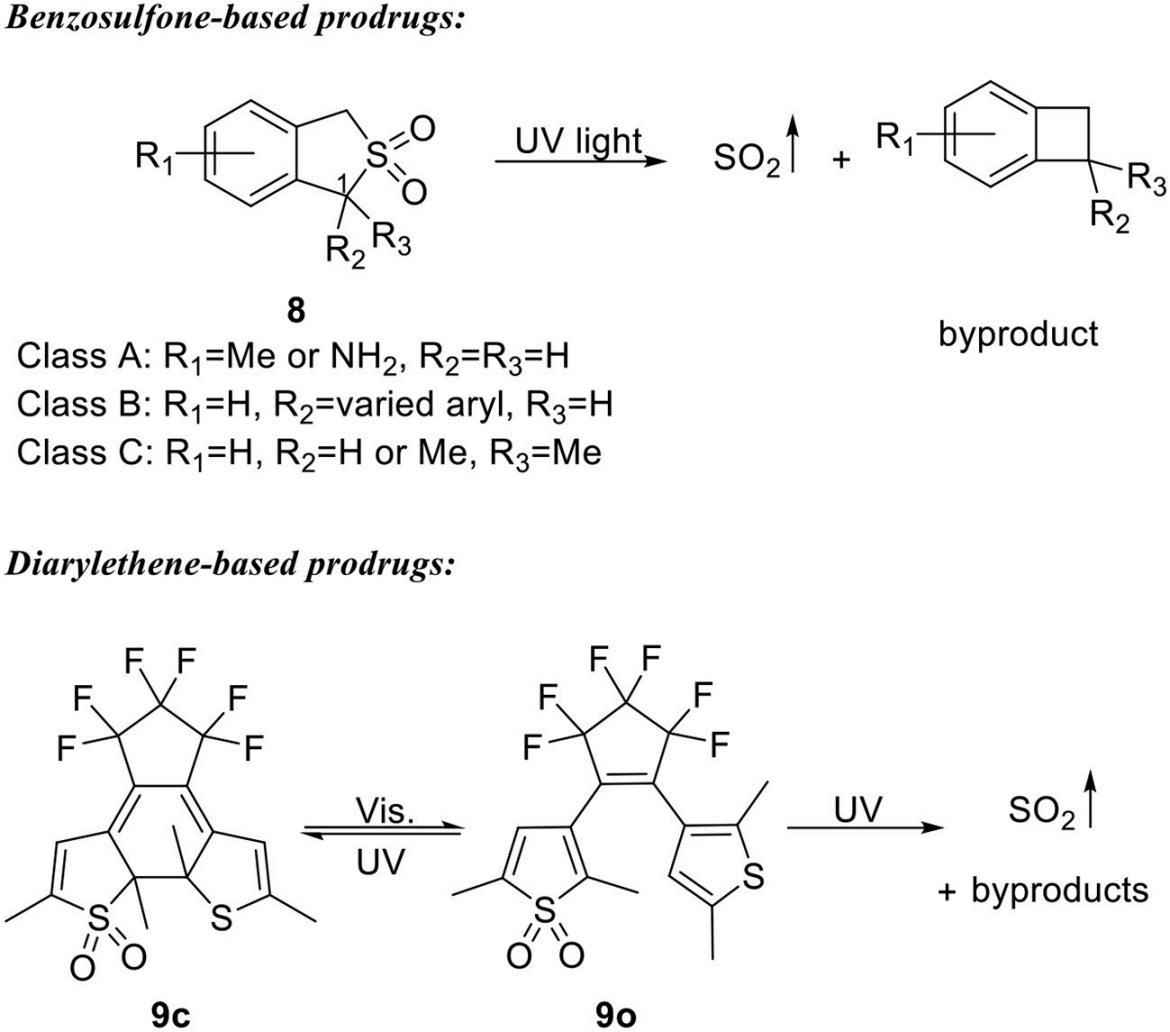
UV-triggered SO_2_ prodrugs.

1-Phenyl-benzosulfone was found to be a more stable form of 1-phenyl-benzosultine (**6**) (Malwal et al., 2013). To develop UV light-triggered SO_2_ prodrugs, Chakrapani's group further exploited the stability of benzosulfones (Malwal and Chakrapani, 2015). Three classes of benzosulfone were designed with varied substitution groups on the 1-position and the aryl ring (**8**, Scheme 3). A total of 17 compounds were synthesized and subjected to UV irritation in pH 7.4 buffered solution (30–40% MeCN or EtOH in PBS). All tested compounds successfully released SO_2_ within 10 min with yields ranging from 12 to 90%. Fifteen compounds gave yields of more than 93% after 60 min. Structure-activity analysis showed that electron-donating groups on the aryl ring and substitutions on 1-position both have a promoting effect on SO_2_ release, possibly through stabilizing the radical intermediates. No biological applications of these prodrugs have been reported so far.

Sumaru and Uchida's group later reported another UV light triggered SO_2_ donor based on a dithienylethene structure and demonstrated its application in inducing cell death “on demand” (Scheme 3) (Kodama et al., 2015). Close-ring compound **9c** was found to be stable upon heating up to 70°C. While upon visible light (λ > 480 nm) or UV light (300 nm < λ <365 nm) irradiation, **9c** undergoes cycloreversion reaction to form open-ring isomer **9o**. Further irradiation by UV light (300 nm < λ <365 nm) of **9o** would lead to both SO_2_ gas formation and cyclization reaction to form **9c**. Therefore, SO_2_ gas can be stored in the form of **9c** as a thermally stable reagent and be released upon UV irradiation. Compounds **9c** and **9o** were then coated on substrates as thin layers and tested for cell death-inducing effect. NIH/3T3 and MDCK cells were disseminated on the thin layers and light at 365 or 436 nm were used in a striped pattern. It was found that 365 nm light would induce cell damage and detachment from the thin layers of both **9c** and **9o** in the patterned area, demonstrating an on-demand feature of killing cells.

### Hydrolysis-based SO_2_ prodrugs

**Scheme 4:**
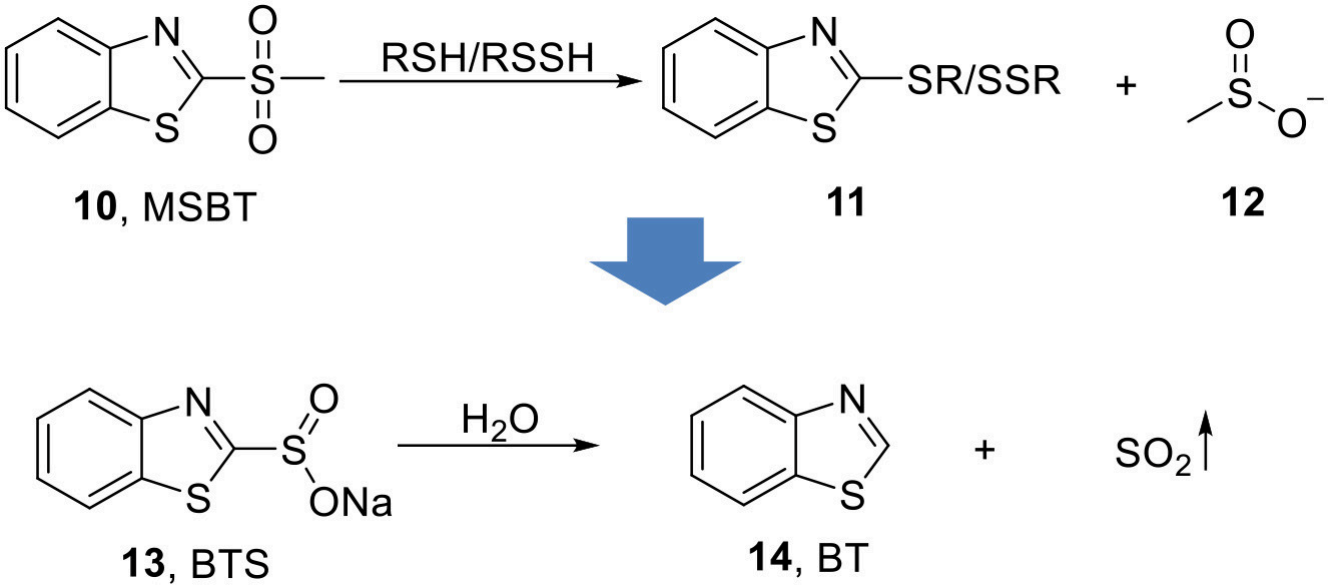
Hydrolysis-based SO_2_ prodrug.

Xian's group later reported sodium benzothiazole sulfinate (**13**, BTS, Scheme 4) as a water-soluble prodrug features slow-release of SO_2_ (Day et al., 2016). The inspiration came from their previous finding that methyl sulfonyl benzothiazole (**10**, MSBT) selectively blocks protein thiols through electrophilic substitution on the C2 position of benzothiazole (**14**, BT), and produce methyl sulfinic acid (**12**) as a byproduct (Scheme 4) (Zhang et al., 2012). Therefore, replacing the methyl sulfone group with a sulfonate group would allow BTS to undergo self-elimination and to release SO_2_ and benzothiazole upon hydrolysis. This idea was well-supported by experimental results. BTS was obtained as crystalline solid through basic hydrolysis of benzothiazole methyl sulfinic ester. As a salt, it has solubility as high as 100 mM in water. Production of SO_2_ through decomposition of BTS and formation of BT was monitored by HPLC. The release of SO_2_ was shown to be second-order that the rate depends on both pH (proton concentration) and concentration of BTS itself. At pH 7.4 and 37°C, 0.4 mM BTS would slowly release SO_2_ with a half-life of about 13 days. Lower pH would promote SO_2_ release that at pH 4, the same concentration of BTS has a half-life of only 7.5 min. The SO_2_ release was further confirmed using a reported ratiometric fluorescent probe (Mito-Ratio-SO_2_) (Xu et al., 2015). This prodrug was then applied in a vasorelaxation experiment using rat aorta rings. 250 μM−4 mM of BTS was applied to aorta rings and demonstrated dose-dependent relaxation effect. When compared with SO_2_ gas, the same concentration of BTS (2 mM) showed a weaker but longer-lasting vasorelaxant effect, while SO_2_ gas has a stronger but shorter effect. Such results are consistent with the slow releasing property of BTS compared with SO_2_ gas.

### Click reaction-based SO_2_ prodrugs

**Scheme 5:**
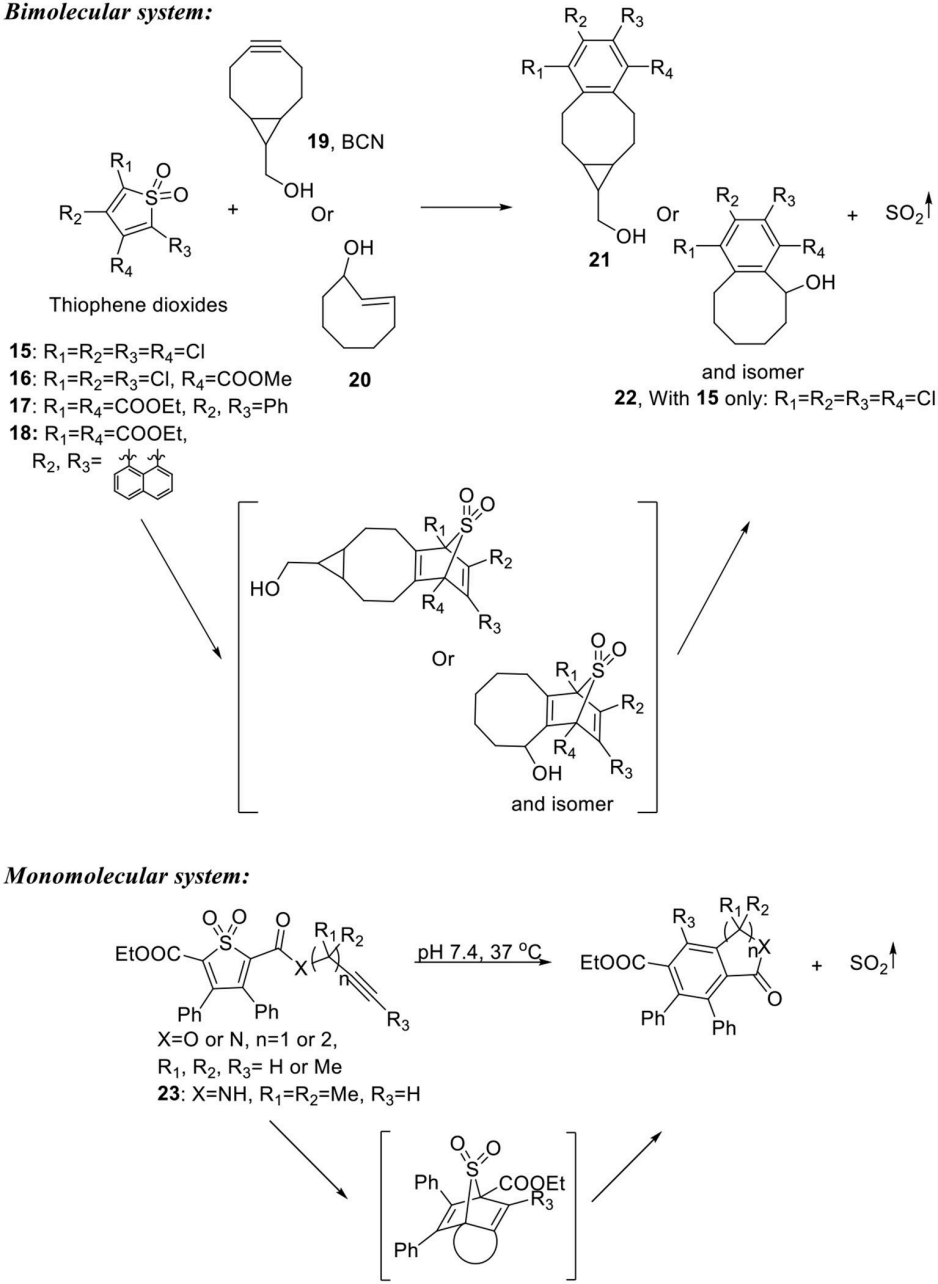
Click-reaction-based SO_2_ prodrugs.

Inspired by their previous work developing CO prodrugs, Wang's group developed click-reaction-based SO_2_ donors and prodrugs (Wang D. et al., 2014; Ji et al., 2017; Wang W. et al., 2017). A bimolecular system consisted of a tetra-substituted thiophene dioxide with a low LUMO and a strained alkyne/alkene with a high HOMO was first developed as a proof-of-concept (Scheme 5). The reactants would first undergo inverse electron-demand Diels-Alder reaction to form an adduct intermediate. Then one molecule of SO_2_ would be released through cheletropic reaction to restore aromaticity. Four thiophene dioxides (**15**–**18**) with varied electron withdrawing groups were synthesized through oxidation of the corresponding thiophene. The compounds were reacted with bicyclononyne (**19**, BCN) or *trans*-cyclooctene (**20**) under room temperature or 37°C. SO_2_ was successfully released from the reaction mixture and detected using Ellman's reagent as a probe. The cheletropic products (**21** and **22**) were also isolated and identified, confirming the proposed reaction mechanism. The release rate was monitored by UV absorption change as the reaction proceeds. Second order reaction rate constants (*k*_2_) ranging from 0.01 to 1.50 M^-1^s^−1^ were obtained at room temperature or 37°C. It is worth noticing that one of the thiophene dioxide (**18**, Scheme 5) was found to be self-reporting. When reacted with BCN, the cheletropic product formed after SO_2_ release has a cyan fluorescence that allows for a real-time monitor of SO_2_ release. However, it will be difficult to apply this prodrug system in a biological environment, since reaching desired release rate would require local concentration control of both components, which has complicated reaction kinetics and does not allow separate controls of dosage and release rate.

To overcome these problems, Wang's group carried the “click-and-release” strategy further by combining the diene (thiophene dioxide) and dienophile (alkyne) into one single molecule (Ji et al., 2017). They reasoned that intramolecular cycloaddition reaction will be entropy-favored and therefore allow the compounds to release SO_2_ under mild conditions. Six compounds were synthesized with varied tether structures while retaining the same substituted thiophene dioxide structure (Scheme 5). By manipulating the tether length, substituents on the tether, and electronic properties of the alkyne moiety, release half-lives were tuned from about 10 min to more than 10 days. It was shown that solvent also played a role in the reaction kinetics that SO_2_ release was faster in mixed aqueous solution than in pure MeCN, possibly due to the ability of the aqueous solvent to accelerate Diels-Alder reactions as described previously (Breslow, 2002). Compound **23** with a half-life of 0.28 h was selected for biological tests. It was applied in a DNA cleavage assay and showed SO_2_-like DNA-cleaving activity. In cell imaging experiments, intracellular SO_2_ release in Raw264.7 cells treated with **23** was successfully detected by a reported bisulfite probe (Sun et al., 2014).

### Esterase-sensitive SO_2_ prodrugs

**Scheme 6:**
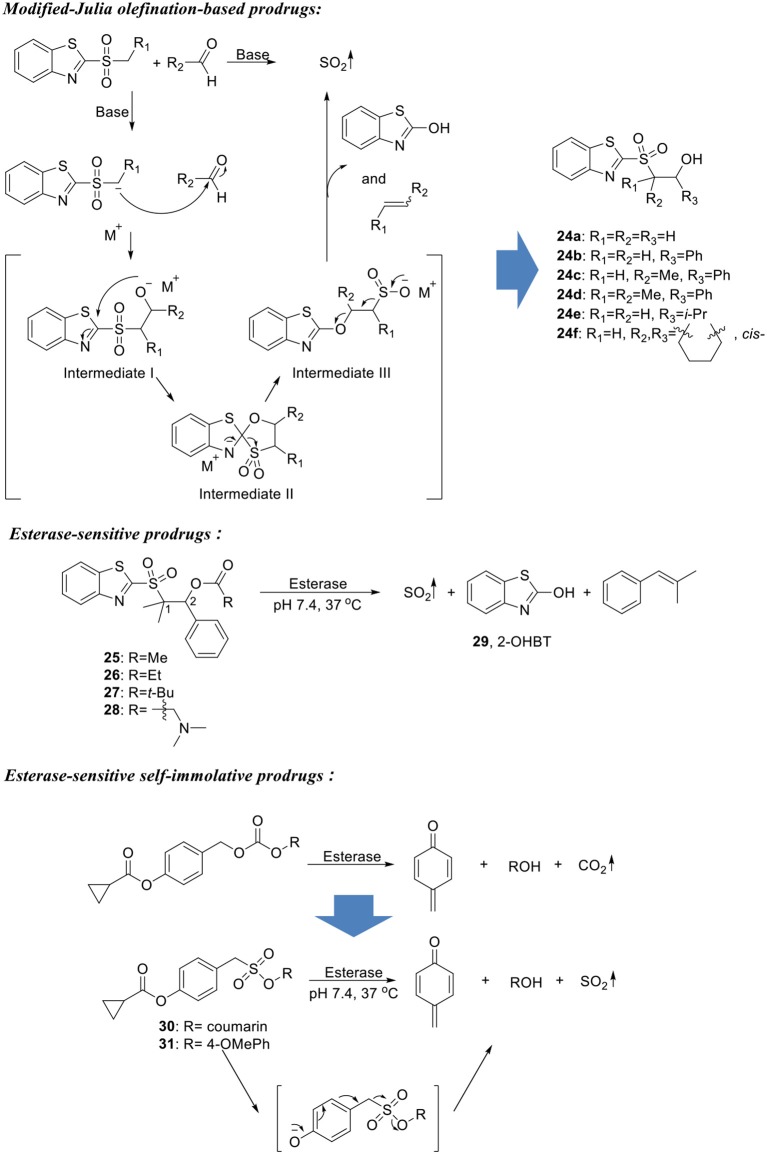
Esterase-sensitive SO_2_ prodrugs.

Recently, Wang's group developed an SO_2_ delivery platform that enables an enzyme-triggered SO_2_ release mechanism (Scheme 6) (Wang and Wang, 2017). The design was based on an intermediate structure formed in modified-Julia olefination reaction. SO_2_ was trapped in a β-alkoxysulfone molecule capped with an ester group. Upon esterase hydrolysis under physiological conditions (pH 7.4, 37°C), the compound would undergo addition-elimination reaction at the C2 position of the BT ring to form a sulfinic acid intermediate, which in turn generates SO_2_ upon spontaneous elimination (Scheme 6). To validate this design, six compounds (**24a**–**24f**) with free hydroxyl group were designed and synthesized with varied substitution pattern on the 1- and 2- positions of β-alkoxysulfone. 2-OHBT (**29**) as reaction byproduct was used to monitor the reaction process using HPLC. Four of the compounds (**24b**–**24e**) successfully generated SO_2_ upon dissolution in pH 7.4 10% DMSO/PBS solution at 37°C, with half-lives ranging from 2 min to 1.5 h. Two fast-releasing compounds (**24c** and **24d**) were selected for ester modification. Compound **24d** showed very good stability under physiological conditions and was further decorated with different ester groups. Reaction rate tuning was achieved by taking advantages of their esterase sensitivity. Four prodrugs (**25**–**28**) were synthesized and indeed showed varied SO_2_ release rates in presence of porcine liver esterase (PLE). It is worth noticing that one of the compounds with an α-dimethyl amino group (**28**) showed greatly improved solubility in aqueous media and a high esterase sensitivity. This compound was then applied in cell imaging studies and demonstrated much higher efficiency delivering SO_2_ to HeLa cells compared with bisulfite salt. It is envisioned that by modifying the free hydroxyl group of **24d**, various triggering mechanisms can be achieved. Therefore, such a prodrug strategy can provide a platform to develop tailored SO_2_ prodrugs to cater to different research needs.

Toward the same end, Chakrapani's group later reported another series of esterase-sensitive SO_2_ donors inspired by the decomposition of carbonate (Scheme 6) (Pardeshi et al., 2018). In their design, an esterase-sensitive moiety (cyclopropyl ester) was connected to a sulfonate moiety through a self-immolative linker, which would give SO_2_ upon esterase hydrolysis and subsequent cleavage of the C-S bond (Scheme 6). As a proof-of-concept, compound **30** with coumarin as the R group was synthesized and tested using HPLC. At 37°C in pH 7.4 PBS, 80% conversion was obtained with a half-life of ~4 min in the presence of 1 unit/mL PLE. While in the absence of esterase, compound **30** was shown to be stable under physiological conditions. To further test the effect of leaving group on SO_2_ delivery, 11 compounds with different R groups and pKa values of corresponding alcohols were synthesized. However, significant hydrolysis of sulfonate was observed in several compounds, leading to a compromised yield of SO_2_. The utility of such SO_2_ prodrugs in the biological system was demonstrated by fluorescent cell imaging assays using compound **31** as a representative. Compound **31** treated A549 cells turned on the strong fluorescence of a sulfite probe, which is in good accordance with its high SO_2_ release ability. Moreover, the prodrug at a much lower concentration (25 μM) gave similar fluorescence intensity when compared with NaHSO_3_ (200 μM), indicating a higher capacity of delivering SO_2_ into cells.

## Discussion

As mentioned above, some of the prodrugs have already been applied in relevant studies as research tools to investigate SO_2_'s mechanism of action, and as a convenient SO_2_ source in the validation of sulfite/bisulfite probes. However, despite the huge therapeutic potential of SO_2_ as a new member of the gasotransmitter family, none of the currently reported prodrugs has been applied in animal models. More detailed studies on the pharmaceutical properties of these prodrugs would be required for future applications. In this section, we give an overview of existing problems and possible solutions for current prodrugs.

Firstly, as a general requirement for therapeutic agents, the aqueous solubility of the prodrugs is important for their biological application. Some of the prodrugs containing large aromatic moieties may have a limited potential toward such an end. Besides, by losing a sulfone/sulfinic group, some of the prodrugs may yield byproducts with lower water solubility. To improve water solubility, attaching hydrophilic moieties on the core structures would be most feasible. Large hydrophilic groups, such as polyethylene glycol (PEG), may improve water solubility and change the pharmacokinetics properties such as circulation time and clearance rate. A balanced lipophilicity and hydrophilicity should be reached to allow transmembrane delivery when intracellular activation is needed. Secondly, since byproducts are inevitable in the release process, it is crucial that the effects of byproducts be carefully considered to avoid misinterpretation of the results. Characterization of major byproducts is necessary. Moreover, when using UV-triggered prodrugs, the UV light exposure time may also need to be well-controlled to avoid biomaterial damage and further complication of the result interpretation. Thirdly, the therapeutic application would require balanced drug metabolism and pharmacokinetics (DMPK) properties. Prodrugs with slower release rates will also require higher stability in the biosystem without metabolism or excretion, while prodrugs with higher release rates need quicker clearance to avoid byproduct build-up and toxicity. The manipulation of DMPK properties can be addressed through chemical modification or formulation. As mentioned above, modifications such as PEG, polysaccharides, carrier (nanoparticles, liposomes, micelles, etc.,), can all contribute to different circulation behavior (Yoo et al., 2010).

## Conclusion

Various SO_2_ donors and prodrugs have been developed and have shown greater potency for therapeutic applications compared with traditional methods of delivering SO_2_. For example, thiol-triggered prodrugs are well-adopted as two-prong antibacterial reagents; UV light-sensitive prodrugs allow for localized release of SO_2_ gas to induce cell death; esterase-sensitive prodrugs deliver SO_2_ into the cells with higher efficiency and specificity. Besides afore-mentioned applications, such SO_2_ donors and prodrugs may as well serve as tools for exploring the mechanism of SO_2_'s biological functions. SO_2_ prodrugs with varied release rates would allow studies of SO_2_'s biological effect in greater detail and suit personalized prescription requirement in potential therapeutic applications. Triggered release mechanism should improve the delivery efficiency of SO_2_ to target cells/tissues/organs, and lower required dosage to achieve desired effect. Controllable release of SO_2_ should facilitate studies on the crosstalk of SO_2_ with other gasotransmitters and drugs in a defined manner. Nevertheless, the current prodrugs suffer more or less from problems such as low release efficiency, low drug loading efficiency, and poor water solubility. As medicinal chemists are most concerned with, the chemical properties of these prodrugs should be optimized to allow good pharmacokinetics performance. Moreover, the components/byproducts of the prodrug system should also be taken into consideration to minimize complications of SO_2_'s effect as well as side effects on the biological system. Future research in this area should better address these problems. Possible approaches could be using chemical modifications and drug delivery systems to improve the properties of current prodrugs. According to the reported biological and pathological roles of SO_2_, it is also important to develop SO_2_ donors with easily-modified tissue specificity (e.g., cardiovascular system specificity). Therefore, developing biomarker-conjugated SO_2_ prodrug systems should be of great biological and therapeutic interests to researchers in this area.

## Author contributions

All authors listed have made a substantial, direct and intellectual contribution to the work, and approved it for publication.

### Conflict of interest statement

The authors declare that the research was conducted in the absence of any commercial or financial relationships that could be construed as a potential conflict of interest.
